# Tolerability of dose-escalating early postoperative intraperitoneal chemotherapy in colorectal cancer with peritoneal disease: EFFIPEC single-arm phase I study

**DOI:** 10.1093/bjsopen/zrag121

**Published:** 2026-10-05

**Authors:** Elinor Bexe Lindskog, Lana Ghanipour, Gabriella Jansson Palmer, Dan Asplund, Caroline Nordenvall, Helgi Birgisson, Ingvar Syk, Torbjörn Swartling, Per J Nilsson, Jan-Erik Frödin, Victor Verwaal, Wilhelm Graf, Peter Cashin

**Affiliations:** Department of Surgery, Institute of Clinical Sciences, Sahlgrenska Academy, University of Gothenburg, Gothenburg, Sweden; Department of Surgery, Sahlgrenska University Hospital, Region Västra Götaland, Gothenburg, Sweden; Department of Surgical Sciences, Uppsala University, Uppsala, Sweden; Department of Surgery and Urology, Uppsala University Hospital, Uppsala, Sweden; Department of Molecular Medicine and Surgery, Karolinska Institutet, Stockholm, Sweden; Department of Surgery, Institute of Clinical Sciences, Sahlgrenska Academy, University of Gothenburg, Gothenburg, Sweden; Department of Surgery, Sahlgrenska University Hospital, Region Västra Götaland, Gothenburg, Sweden; Department of Molecular Medicine and Surgery, Karolinska Institutet, Stockholm, Sweden; Department of Surgical Sciences, Uppsala University, Uppsala, Sweden; Department of Surgery and Urology, Uppsala University Hospital, Uppsala, Sweden; Institution of Clinical Science, Malmö, Lund University, Malmö, Sweden; Department of Surgery, Institute of Clinical Sciences, Sahlgrenska Academy, University of Gothenburg, Gothenburg, Sweden; Västerås County Hospital, Department of Surgery, Västerås, Sweden; Department of Molecular Medicine and Surgery, Karolinska Institutet, Stockholm, Sweden; Department of Oncology and Pathology, Karolinska Institutet, Stockholm, Sweden; Institution of Clinical Science, Malmö, Lund University, Malmö, Sweden; Department of Surgery, Skåne University Hospital, Malmö, Sweden; Department of Surgical Sciences, Uppsala University, Uppsala, Sweden; Department of Surgical Sciences, Uppsala University, Uppsala, Sweden; Department of Surgery and Urology, Uppsala University Hospital, Uppsala, Sweden

**Keywords:** peritoneal metastases, cytoreductive surgery, HIPEC, EPIC, clinical trial

Colorectal peritoneal metastases remain a challenging clinical setting in which the added value and optimal regimen of intraperitoneal chemotherapy are unresolved. PRODIGE 7^1^ questioned 30-minute single-agent oxaliplatin hyperthermic intraperitoneal chemotherapy (HIPEC) after cytoreductive surgery (CRS), whereas subsequent observational data^2^ suggest that regimen and drug selection may be important. The EFFIPEC phase I study was designed to test whether intensified oxaliplatin/irinotecan HIPEC combined with condensed early postoperative intraperitoneal chemotherapy (EPIC) could be delivered safely before randomized evaluation^3^. This phase I study aimed to establish the maximum tolerated dose of 1-day intraperitoneal 5-fluorouracil (5-FU) EPIC after CRS and intensified HIPEC while describing early postoperative morbidity relevant to further trial development.

EFFIPEC phase I was a Swedish multicentre, single-arm, 3 + 3 dose-escalation trial registered at EudraCT (2020-005210-18). Eligible patients were aged 18–75 years, had colorectal peritoneal metastases judged suitable for complete CRS/HIPEC, Eastern Cooperative Oncology Group performance status 0–2, adequate organ function, and no extraperitoneal metastases. After CRS, patients received intravenous 5-FU 400 mg/m^2^ and calcium folinate 60 mg/m^2^, followed by 30-minute HIPEC with oxaliplatin 360 mg/m^2^ and irinotecan 360 mg/m^2^ at 41–42°C; this dual-drug dose was based on previous pharmacological studies^4^. At the end of CRS/HIPEC, 5-FU EPIC was delivered at planned dose levels of 250, 400, 550, 700, and 850 mg/m^2^ dissolved in 400 ml saline and perfused through two intraperitoneal drains (200 ml in each drain). The drains were clamped for at least 16 hours after drug instillation. Dose-limiting toxicity was defined as Clavien–Dindo grade IIIb–IV morbidity in at least three of six patients per dose level. Granulocyte colony-stimulating factor was administered on postoperative days 4–8. The dose escalation scheme, eligibility criteria, baseline characteristics, surgical characteristics, study flowchart, and postoperative white blood cell count are provided in *Tables S1–S4* and *Figs S1* and *S2*.

Between May 2021 and November 2022, 28 patients were screened and 17 were included; 15 completed protocol treatment. The median age was 67 years, nine patients were women, the median peritoneal cancer index was 6, and the median operating time was 350 min. Three patients completed full treatment at each planned dose level. No maximum tolerated dose was reached. Three Clavien–Dindo grade IIIb–IV events occurred: wound dehiscence with bleeding at 250 mg/m^2^, anastomotic leakage at 700 mg/m^2^, and colonic perforation at 850 mg/m^2^ (*Table 1*). No grade V event occurred, no dose level required expansion to six patients because of dose-limiting toxicity, and serious morbidity did not increase with dose escalation. Grade IIIa events comprised intra-abdominal infection, pulmonary embolism, pleural effusion, and urinary leakage. Neutropenia was the main toxicity despite prophylactic bone marrow stimulation, with grade II–IV neutropenia in seven of 17 included patients; the postoperative white blood cell pattern is shown in *Fig. S2*. Neoadjuvant chemotherapy before CRS/HIPEC was not statistically associated with lower absolute neutrophil counts or neutropenia, although neutropenia was numerically more frequent after neoadjuvant treatment.

**Table 1:** Postoperative Clavien–Dindo grade IIIa–IV complications after cytoreductive surgery/hyperthermic intraperitoneal chemotherapy per dose escalation level of 1-day early postoperative intraperitoneal chemotherapy (5-fluorouracil)

	Dose escalation level				
	Level −1 (250 mg/m^2^; *n* = 3)	Level 0 (400 mg/m^2^; *n* = 3)	Level 1 (550 mg/m^2^; *n* = 5)	Level 2 (700 mg/m^2^; *n* = 3)	Level 3 (850 mg/m^2^; *n* = 3)
**Grade IIIa**	1 (33 %)	3 (100 %)	1 (33 %)	0 (0 %)	0 (0 %)
Pulmonary embolism	0	0	1	0	0
Intra-abdominal infection	0	2	0	0	0
Pleural effusion	1	0	0	0	0
Urinary leakage	0	1	0	0	0
**Grade IIIb**	0 (0 %)	0 (0 %)	0 (0 %)	1 (33 %)	1 (33 %)
Anastomotic leakage	0	0	0	1	0
Colonic perforation	0	0	0	0	1
**Grade IVa**	1 (33 %)	0 (0 %)	0 (0 %)	0 (0 %)	0 (0 %)
Wound dehiscence with bleeding	1	0	0	0	0
Grade of neutropenia*	1 (33 %)	1 (33 %)	1 (33 %)	2 (67 %)	2 (67 %)
Grade II	1	1	0	0	1
Grade III	0	0	1	0	1
Grade IV	0	0	0	2	0

Values show the number of patients in each group. *Neutropenia was an expected side effect of the treatment and not considered a dose-limiting toxicity.

Intensified oxaliplatin/irinotecan HIPEC combined with 1-day 5-FU EPIC was feasible after CRS for colorectal peritoneal metastases. The highest EPIC dose tested, 850 mg/m^2^, was therefore selected for the ongoing randomized phase III EFFIPEC trial. The principal toxicity signal was neutropenia, consistent with previous experience of oxaliplatin-based HIPEC^5^, where neutropenia has not been associated with increased surgical morbidity. Despite prophylactic granulocyte colony-stimulating factor, neutropenia remained relatively common, occurring in two of three patients at each of the two highest dose levels (*Table 1*). This toxicity will require continued monitoring in the ongoing phase III trial. These findings should be interpreted strictly as dose-finding and feasibility data. Efficacy, recurrence outcomes, and comparative safety are being evaluated in the randomized phase III component of EFFIPEC.

## Supplementary Material

zrag121_Supplementary_Data

## Data Availability

The data underlying this article will be shared on reasonable request to the corresponding author. The purpose of the request will be evaluated in relation to regulatory and ethical approvals before release.

## References

[1] Quénet F, Elias D, Roca L, Goéré D, Ghouti L, Pocard M et al Cytoreductive surgery plus hyperthermic intraperitoneal chemotherapy *versus* cytoreductive surgery alone for colorectal peritoneal metastases (PRODIGE 7): a multicentre, randomised, open-label, phase 3 trial. Lancet Oncol 2021;22:256–266 10.1016/S1470-2045(20)30599-4 33476595

[2] Fisher OM, Brown C, Esquivel J, Larsen SG, Liauw W, Alzahrani NA et al Hyperthermic intraperitoneal chemotherapy in colorectal cancer. BJS Open 2024;8:zrae050 10.1093/bjsopen/zrae017 PMC11081075 38722737

[3] Ghanipour L, Jansson Palmer G, Nilsson PJ, Nordenvall C, Frödin JE, Bexe Lindskog E et al Efficacy of hyperthermic intraperitoneal chemotherapy in colorectal cancer: a phase I and III open-label randomized controlled registry-based clinical trial protocol. PLoS One 2024;19:e0294018 10.1371/journal.pone.0294018 PMC10911585 38437211

[4] Elias D, Raynard B, Bonnay M, Pocard M. Heated intra-operative intraperitoneal oxaliplatin alone and in combination with intraperitoneal irinotecan: pharmacologic studies. Eur J Surg Oncol 2006;32:607–613 10.1016/j.ejso.2006.03.004 16621432

[5] Cashin PH, Ghanipour L, Enblad M, Morris DL. Neutropenia in colorectal cancer treated with oxaliplatin-based hyperthermic intraperitoneal chemotherapy: an observational cohort study. World J Gastrointest Oncol 2020;12:549–558 10.4251/wjgo.v12.i5.549 PMC7235182 32461786

