# Supplementary material for: Tolerability of dose-escalating early postoperative intraperitoneal chemotherapy in colorectal cancer with peritoneal disease: EFFIPEC single-arm phase I study
Source: BJS Open. 2026 Oct 5;10(5):zrag121. doi: 10.1093/bjsopen/zrag121 (PMC13637039; doi:10.1093/bjsopen/zrag121)
Supplement: Supplementary file 1 [file zrag121_supplementary_data.docx]

**Tolerability of Dose-escalating Early Postoperative Intraperitoneal Chemotherapy in Colorectal Cancer with Peritoneal Disease – the EFFIPEC Single-arm Phase I study**

Elinor Bexe Lindskog^1,2^, Lana Ghanipour^3,4^, Gabriella Jansson Palmer^5^, Dan Asplund^1,2^, Caroline Nordenvall^5^, Helgi Birgisson^3,4^, Ingvar Syk^6^, Torbjörn Swartling^1,7^, Per J Nilsson^5^, Jan-Erik Frödin^8^, Victor Verwaal^6,9^, Wilhelm Graf^3^ and Peter Cashin^3,4^

1. Department of Surgery, Institute of Clinical Sciences, Sahlgrenska Academy, University of Gothenburg, Gothenburg, Sweden
2. Region Västra Götaland, Sahlgrenska University Hospital, dept of Surgery, Gothenburg, Sweden

3. Department of Surgical Sciences, Uppsala University, Uppsala, Sweden
4. Department of Surgery and urology, Uppsala University Hospital, Uppsala, Sweden
5. Department of Molecular Medicine and Surgery, Karolinska Institutet, Stockholm, Sweden

6. Institution of clinical science, Malmö, Lund University, Malmö, Sweden

7. Västerås County Hospital, dept of Surgery, Västmanlands Region, Västerås, Sweden
8. Department of Oncology and Pathology, Karolinska Institutet, Stockholm, Sweden
9. Department of Surgery, Skåne University Hospital, Malmö, Sweden

**Supplementary Material**

Table of contents:

Figure S1 – Flowchart	p 2

Figure S2 – Leukocyte levels postoperatively	p 3

Table S1 – Dose escalation scheme	p 4

Table S2 – Inclusion/exclusion criteria	p 5

Table S3 – Patient and tumor characteristics	p 6

Table S4 – Surgical characteristics	p 7

Supplementary Results	p 8

Figure S1: Flowchart of the EFFIPEC phase I trial

Figure S2: White blood cell counts postoperatively between patients with and without leucopenia. The grey time period marks the days in which patient receive prophylactic granulocyte colony stimulating factor.

Table S1: Planned 3+3 dose escalation scheme

| Level | Oxaliplatin IP | Irinotecan  IP | 5-FU IP | 5-FU IV | Calcium folinate IV |
| --- | --- | --- | --- | --- | --- |
| Type | 30-min HIPEC | 30-min HIPEC | 16-hour EPIC | Bolus | Bolus |
| -1 | 360 mg/m^2^ | 360 mg/m^2^ | 250 mg/m^2^ | 400 mg/m^2^ | 60mg/m^2^ |
| 0 | 360 mg/m^2^ | 360 mg/m^2^ | 400 mg/m^2^ | 400 mg/m^2^ | 60mg/m^2^ |
| 1 | 360 mg/m^2^ | 360 mg/m^2^ | 550 mg/m^2^ | 400 mg/m^2^ | 60mg/m^2^ |
| 2 | 360 mg/m^2^ | 360 mg/m^2^ | 700 mg/m^2^ | 400 mg/m^2^ | 60mg/m^2^ |
| 3 | 360 mg/m^2^ | 360 mg/m^2^ | 850 mg/m^2^ | 400 mg/m^2^ | 60mg/m^2^ |

Table S2: Inclusion and exclusion criteria.

| INCLUSION CRITERIA | EXCLUSION CRITERIA |
| --- | --- |
| 1. Written informed consent prior to any study specific procedures.  2. ECOG Performance Status Score 0,1 or 2  3. Adequate kidney, liver, bone marrow function according to laboratory tests  4. For females of childbearing potential, a negative pregnancy test must be documented  5. Age 18-75  6. Colorectal cancer with peritoneal metastases  7. All patients deemed eligible for CRS and HIPEC according to clinical routine management during a multidisciplinary HIPEC board at each hospital can be included. | 1. Previous severe toxicity/allergic reactions to systemic chemotherapy agents oxaliplatin or irinotecan or 5-fluorouracil  2. Unable to tolerate intensified HIPEC treatment due to comorbidity  3. Metastasis other than peritoneum  4. Pregnant or lactating (nursing) women  5. Active infections requiring antibiotics  6. Active liver disease with positive serology for active hepatitis B, C, or known HIV  7. Concurrent administration of any cancer therapy other than planned study treatment within 4 weeks prior to and up to 4 weeks after study treatment  8. Incomplete cytoreduction defined as completeness of cytoreduction score 1-3  9. Histopathology of other origin than colorectal cancer |

| Table S3: Patient and tumor characteristics of all patients enrolled in EFFIPEC phase I (*n*=17). | | | |
| --- | --- | --- | --- |
| **Age (years)** |  |  |  |
| Median (IQR) |  | 67 (62-69) |  |
| **Gender, *n*** |  |  |  |
| Female |  | 9 (53%) |  |
| Male |  | 8 (47%) |  |
| **Primary tumor location, *n*** |  |  |  |
| Right sided |  | 9 (53%) |  |
| Left sided |  | 7 (41%) |  |
| Rectum |  | 1 (6%) |  |
| **Primary tumor T-stage, *n*** |  |  |  |
| T3 |  | 4 (24%) |  |
| T4a |  | 10 (59%) |  |
| T4b |  | 3 (18%) |  |
| **Primary tumor N-stage, *n*** |  |  |  |
| N0 |  | 2 (12%) |  |
| N1 |  | 6 (35%) |  |
| N2 |  | 9 (53%) |  |
| **ECOG, *n*** |  |  |  |
| 0 |  | 15 (88%) |  |
| 1 |  | 2 (12%) |  |
| **Peritoneal Metastases** |  |  |  |
| Synchronous |  | 8 (47%) |  |
| Metachronus |  | 9 (53%) |  |
| **Prior chemotherapy** |  |  |  |
| Neoadjuvant before prior primary tumor resection |  | 6 (35%) |  |
| Adjuvant after prior primary tumor resection |  | 9 (53%) |  |
| Neoadjuvant prior to CRS and HIPEC |  | 7 (41%) |  |
| Abbreviations; PM, peritoneal metastases, PCI: peritoneal cancer index. | | | |

| Table S4: Surgical Characteristics |  |  |
| --- | --- | --- |
| **OR time and bleeding – median (IQR)** |  |  |
| OR time in minute | 350 (270-430) |  |
| Blood loss in ml | 500 (200-1200) |  |
| **Intraoperative PCI, median (range)** | 6 (2–17) |  |
| **Organs resected – n (%)** |  |  |
| Colon | 8 (47%) |  |
| Rectum | 8 (47%) |  |
| Small bowel | 8 (47%) |  |
| Ovary resection in nine women | 5 (56%) |  |
| Spleen | 2 (12%) |  |
| Liver resection due to implantation | 3 (18%) |  |
| Uterus resection in nine women | 4 (44%) |  |
| Diaphragm | 2 (12%) |  |
| **Stoma formation – n (%)** |  |  |
| Yes | 9 (53%) |  |
| No | 5 (29%) |  |
| Stoma present prior to and after surgery | 3 (18%) |  |
| **Number of anastomoses – n (%)** |  |  |
| 0 | 8 (47%) |  |
| 1 | 8 (47%) |  |
| 2 | 1 (6%) |  |

**Supplementary Results**

All Clavien-Dindo grade IIIa or more as reported in the main manuscript were identified as serious adverse events according to ICH-GCP definition. There was one event identified as a serious adverse event that did not qualify for Clavien-Dindo grade III+ classification in level 1 (550mg/m^2^). This patient developed high-output stoma leading to prolonged hospital stay due to saline drip which was classified as Clavien-Dindo grade II. There were no kidney failure events. Adjuvant treatment was administered to eight patients. Data on stoma reversals during follow-up were not collected per the study database.
